# Protocol for preparing *Drosophila* genomic DNA to create chromosome-level *de novo* genome assemblies

**DOI:** 10.1016/j.xpro.2024.102974

**Published:** 2024-04-05

**Authors:** Alexis L. Sperling, Daniel K. Fabian, Erik Garrison, David M. Glover

**Affiliations:** 1 University of Cambridge; Department of Genetics, Cambridge CB2 3EH, UK; 2 University of Tennessee Health Science Center, Memphis, TN 38163, USA; 3 Division of Biology and Biological Engineering, California Institute of Technology, Pasadena, CA 91125, USA

**Keywords:** Bioinformatics, Genetics, Genomics

## Abstract

*De novo* genome assemblies are common tools for examining novel biological phenomena in non-model organisms. Here, we present a protocol for preparing *Drosophila* genomic DNA to create chromosome-level *de novo* genome assemblies. We describe steps for high-molecular-weight DNA preparation with phenol or Genomic-tips, quality control, long-read nanopore sequencing, short-read DNA library preparation, and sequencing. We then detail procedures of genome assembly, annotation, and assessment that can be used for downstream comparison and functional analysis.

For complete details on the use and execution of this protocol, please refer to Sperling et al.^1^

## Highlights

• Protocol for high-molecular-weight DNA isolation and quality control
• Workflow for *de novo* genome assembly and quality assessment
• Instructions for *de novo* genome annotation and quality assessment
• Prepared genomes can be used for subsequent functional analysis

## Abstract (editor highlights)

Publisher’s note: Undertaking any experimental protocol requires adherence to local institutional guidelines for laboratory safety and ethics.

## Graphical abstract

**Figure:**
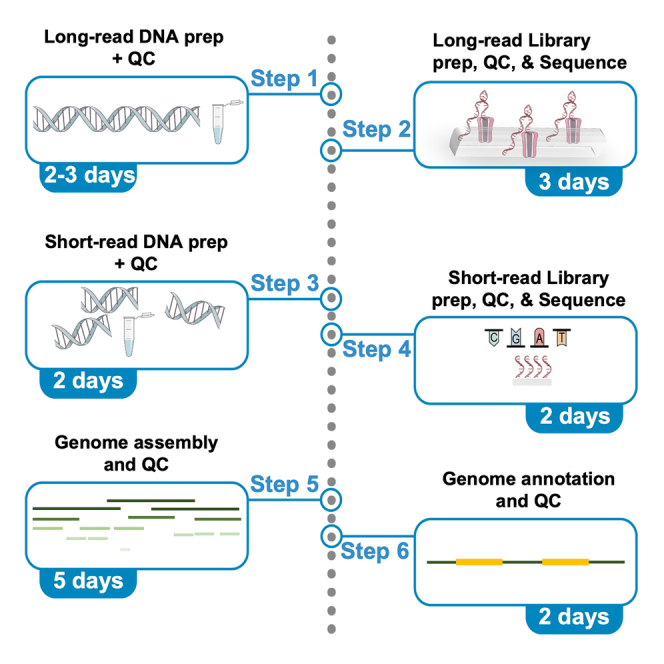

## Before you begin

This protocol was designed for the preparation of high molecular weight genomic DNA from the non-model organism *Drosophila mercatorum*. We prepared two different strains, one sexually reproducing and one parthenogenetic (asexually reproducing). These genomes were compared to each other and to the model organism *Drosophila melanogaster* for quality reassurance and annotation purposes.

The protocol below describes DNA preparation, genome analysis, and quality control measures used for fly genome preparation and comparison. However, it can easily be adapted for genomes of other species. It is important to decide which DNA preparation method might be more suitable for your purpose, given the results you are aiming for. For higher coverage of repetitive regions, the Genomic-tip preparation method works very well. However, a more contiguous genome can be assembled from DNA prepared with the phenol method. The phenol method has the potential risk of carry over that can interfere with certain types of sequencing, namely with Oxford Nanopore Technology (ONT/Nanopore) sequencing, we have added troubleshooting tips on how to avoid this. The Genomic-tip method does not have the phenol carryover risk.

### High molecular weight DNA preparation

It is important to note that high molecular weight DNA is sheared rather easily, therefore care must be taken with handling. Low-bind plastics help prevent shearing of the DNA. It is also best to use wide-bore tips when pipetting solutions containing DNA. Although less ideal, in a pinch it is also possible to cut the ends off your tips to make them wide-bore. It is important not to vigorously shake the DNA containing tubes and never vortex it, unless when specifically indicated.

### Long-read nanopore sequencing

Sequencing protocols evolve rapidly, we therefore recommend referring to the most current protocols recommended by Nanopore.

### Genome assembly, annotation and assessment

The software listed in the key resources table all need to be installed prior to starting the bioinformatics analysis. For each program the installation instructions are provided on the website or associated repository provided in the key resources table. R programming language will need to be installed as well as operating system dependent software, such as Homebrew for macOS.

## Key resources table

**Table:** 

REAGENT or RESOURCE	SOURCE	IDENTIFIER
**Chemicals, peptides, and recombinant proteins**		

SDS	Sigma	L3771
Tris base	Sigma	252859
EDTA	Sigma	E9884
NaCl	Sigma	S9888
Ethanol absolute	Sigma	1009861000
Isopropanol	Sigma	34863
RNAlater stabilization solution	Thermo Fisher Scientific	AM7020
Phenol/chloroform/isoamyl alcohol (25:24:1)	Acros Organics from Fisher Scientific	10308293

**Critical commercial assays**		

RNase A	Thermo Fisher Scientific	EN0531
Proteinase K	Thermo Fisher Scientific	EO0491
Genomic-tip (100/G)	QIAGEN	10243
Genomic-tip buffer set	QIAGEN	13343
Qubit reagents – broad range DNA	Thermo Fisher Scientific	Q33212
Qubit tubes	Thermo Fisher Scientific	Q32856
10X KBB	Sage Science	KB1001
DNA TapeStation reagents	Agilent	5067-5585
Tape	Agilent	5067-5584
Ligation sequencing kit	Nanopore	SQK LSK-109/ SQK LSK-110
Flow cell priming kit	Nanopore	EXP-FLP004-XL
R9 version Spot-ON Flow Cell.	Nanopore	FLO-MIN106D
KAPA HyperPrep kits for NGS DNA library prep	Roche	KK8542
NEBNext Ultra II DNA library prep kit	New England Biolabs	E7645S

**Deposited data**		

*D. mercatorum* raw and analyzed transcriptomics data	Sperling et al.^1^	ENA: PRJEB43100
*D. mercatorum* raw and analyzed genomics data	Sperling et al.^1^	ENA: PRJEB64421
*D. melanogaster* gene names and FBgn numbers	http://flybase.org/	fbgn_NAseq_Uniprot_fb_2020_03.tsv
*D. melanogaster* CDS (reference version 6.35) and Genome (v6.27)		https://www.ncbi.nlm.nih.gov/genome?term=vih&cmd=DetailsSearch
*D. melanogaster* Genome Release 6 plus ISO1 MT	The FlyBase Consortium/Berkeley Drosophila Genome Project/Celera Genomics	https://www.ncbi.nlm.nih.gov/assembly/GCF_000001215.4/

**Experimental models: Organisms/strains**		

*D. mercatorum* wild-type, adult females, freshly eclosed.	Cornell Stock Centre	CSC 15082-1511.00
*D. mercatorum* parthenogenetic, adult females, freshly eclosed.	Cornell Stock Centre	CSC 15082-1525.07

**Software and algorithms**		

wtdbg2 (v2.5)	Ruan et al.^2^	https://github.com/ruanjue/wtdbg2
minimap2 (v2.24)	Li et al.^3^	https://github.com/lh3/minimap2/releases
Samtools	Li et al.^4^	https://www.htslib.org/
Freebayes	Garrison and Marth^5^	https://github.com/freebayes/freebayes
bcftools consensus (v2.18)	Li^6^	https://github.com/samtools/bcftools/releases/
wfmash (v0.6)	Guarracino^7^	https://github.com/waveygang/wfmash/releases
bedtools (v2.18)	Quinlan and Hall^8^	https://github.com/arq5x/bedtools2
meryl (v1.3) and ) and merqury(v1)	Rhie et al.^9^	https://github.com/marbl/meryl
RepeatModeler2 (v2.0.1)	Flynn et al.^10^	https://github.com/Dfam-consortium/RepeatModeler/blob/master/RELEASE-NOTES
RepeatMasker (v4.0.9)		https://www.repeatmasker.org/
Cutadapt (v2.0)	Smit et al.^11^	https://cutadapt.readthedocs.io/en/v2.0/
STAR (v2.7.0e)	Dobin et al.^12^	https://github.com/alexdobin/STAR/releases
BRAKER2	Bruna et al.^13^	https://github.com/Gaius-Augustus/BRAKER
BLASTx (ncbi-blast-2.10.1)	Zhang et al.^14^	https://ftp.ncbi.nlm.nih.gov/blast/documents/blastdb.html
MUMmer (v3.23)	Kurtz et al.^15^	https://mummer.sourceforge.net/
BUSCO (v5.3.2)	Manni et al.^16^	https://busco.ezlab.org/
featureCounts (subread v1.6.3)	Liao et al.^17^	https://rnnh.github.io/bioinfo-notebook/docs/featureCounts.html

**Other**		

Handheld electric pestle mixer		
Plastic pestle		
Water bath		
Centrifuge (refrigerated and room temperature)		
Liquid nitrogen		
Votex		
Metal tongs		
NanoDrop		
Qubit		
Agilent TapeStation		
Pulse field gel electrophoreses apparatus		
Nanopore MinION		

## Materials and equipment

**Table:** SDS buffer

Reagent	Final concentration	Amount
SDS	0.5% (w/v)	0.25 mg
Tris (1 M)	0.200 M	10 mL
EDTA (0.5 M)	0.25 M	25 mL
NaCl_2_ (5 M)	0.250 M	2.5 mL
**Total**	**N/A**	**50 mL**

Note on storage conditions: 21°C (room temperature), maximum storage time 6 months.

### Elution buffer

• 10 mM Tris, pH 8.0 in nuclease free water (0.1 mM EDTA can be included).

Note on storage conditions: made fresh.

## Step-by-step method details

We prepared two *de novo* genomes for two strains of *D. mercatorum* flies using two different high molecular weight DNA preparation methods. One strain is a completely parthenogenetic strain from a newly colonized habitat (Hawaii)^18^ with the DNA prepared using a phenol extraction method. The other strain is sexually reproducing (wild-type) from an ancestral habitat (Brazil) with the DNA prepared using the Genomic-tip (QIAGEN) method.

### High molecular weight DNA preparation with phenol or genomic-tips

#### High molecular weight DNA isolation with phenol

**Timing: 3 days**

The isolation of high molecular weight DNA using the phenol extraction method. This method was a mixed protocol adapted from two other DNA preparation methods.^19^^,^^20^

1. Dissociate Tissue.
  a. Homogenize sample.
    i. Add 150 μL of Sodium Dodecyl Sulfate (SDS) buffer to 1.5 mL tube containing 60 mg of sample (in our case 30–60 flies).
    ii. Break apart sample using a handheld electric pestle mixer for 10 s.
      ***Note:*** This may need to be optimized for different sample types.
    iii. Add 350 μL of SDS buffer to the homogenate.
    iv. Mix by inverting the tube gently 5 times.
    v. Incubate at 37°C for 4 h without agitation.
      ***Note:*** The incubation time can be decreased to 2 h or increased up to 16 h. We had the best preparation with a 4-h incubation.
  b. Eliminate RNA.
    i. Add 5 μL of RNase A (100 mg/mL) to the 1.5 mL tube containing the homogenate.
    ii. Mix by inverting the tube gently 5 times.
    iii. Incubate at 50°C for 2 h without agitation.
  c. Digest Protein.
    i. Add 5 μL of Proteinase K (20 mg/mL).
    ii. Mix by inverting the tube gently 5 times.
    iii. Incubate at 37°C for 2 h without agitation.
2. Isolate DNA.
  a. First extraction.
    i. Add 240 μL of the phenol layer from phenol/chloroform/isoamyl alcohol (25:24:1) to the 1.5 mL tube containing the homogenate. Troubleshooting 1.
    ii. Mix gently for 3 min at 21°C (room temperature).
    iii. Centrifuge at 12,000 × *g* for 10 min.
    iv. Decant supernatant into a new tube.
  b. Second extraction.
    i. Add 240 μL of the phenol layer from phenol/chloroform/isoamyl alcohol (25:24:1) to the 1.5 mL tube containing the supernatant.
    ii. Mix gently for 3 min at 21°C (room temperature).
    iii. Centrifuge at 12,000 × *g* for 10 min.
    iv. Decant supernatant into a new tube.
  c. Third extraction.
    i. Add 240 μL of the chloroform layer from phenol/chloroform/isoamyl alcohol (25:24:1) to the 1.5 mL tube containing the supernatant.
    ii. Mix gently for 3 min at 21°C (room temperature).
    iii. Centrifuge at 12,000 × *g* for 10 min.
    iv. Decant supernatant into a new tube.
  d. Precipitate and isolate DNA.
    i. Add 500 μL of −20°C absolute ethanol.
    ii. Precipitate at −20°C for at least 5 min.
      ***Note:*** This is a potential pause point, where DNA can be stored for 16 h (overnight) at −20°C.
    iii. Remove DNA by spooling with a bent pipette tip.
      ***Note:*** The DNA is visible (Figure 1).
      **Figure 1:**
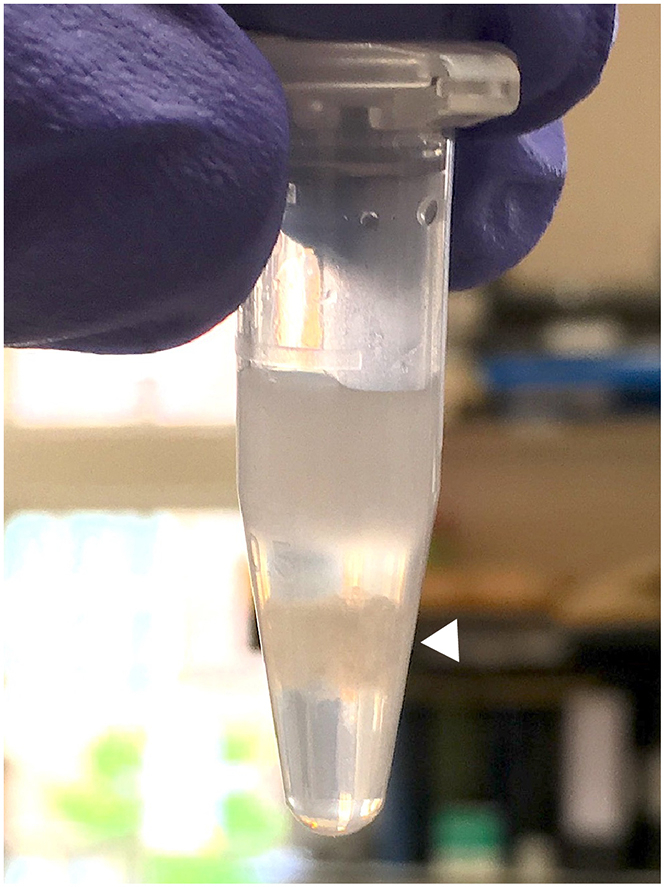
Precipitated genomic DNA The white arrow indicates the precipitated DNA
    iv. Place DNA into a new tube with 80% ethanol.
    v. Centrifuge at 12,000 × *g* for 3 min to remove residual salt.
    vi. Decant the DNA pellet.
    vii. Place tube at 37°C for approximately 30 min (it is important not to leave the DNA on heat longer than necessary). Troubleshooting 2.
    viii. Add 50 μL of elution buffer.
    ix. Leave DNA at 21°C (room temperature) for 16 h (overnight) and then at 4°C for a minimum of 2 days prior to quality assessment and sequencing.
      **CRITICAL:** It is important to not pipette the DNA, when possible, pour the DNA over or use wide-bore, low-bind pipette tips.
      **CRITICAL:** phenol/chloroform/isoamyl alcohol is toxic and should be used in a fume hood with the appropriate protective equipment. Do not use phenol if it has taken on an orange or red color.

#### High molecular weight DNA isolation with QIAGEN genomic-tips

**Timing: 2 days**

This is a modified protocol for high molecular DNA isolation with QIAGEN Genomic-tips.

3. Disrupt cells.
  a. Prepare the lysis buffer by adding 19 μL RNase A to 9.5 mL of Buffer G2.
    i. Mix and setting aside.
  b. Place 60 mg of sample (30–60 flies) in a 1.5 mL tube.
    i. Place 1.5 mL tube with the flies in liquid Nitrogen (N_2_).
      ***Note:*** Use tongs.
    ii. Place pestle tip in liquid N_2_. Troubleshooting 3.
    iii. While the bottom of the tube is in contact (use tongs) with the N_2_ slowly manually grind the flies until broken up.
    iv. Blend the broken-up flies with a handheld electric pestle mixer until a fine powder.
      ***Note:*** For the best results the tube must remain partially submerged in the N_2_ in order to prevent condensation from forming on the sample.
4. Extract and concentrate DNA.
  a. Add 240 μL of lysis buffer to the 1.5 mL tube containing the fly powder and then transfer the lysis-fly solution into a 15 mL tube (on ice).
    i. Repeat 3 times to ensure that all the material is transferred.
    ii. Add the remaining lysis buffer to the tube.
  b. Immediately vortex for 5 s.
  c. Incubate lysate at 37°C for 1 h, gently inverting tube after 30 min.
  d. Digest protein
    i. Add 500 μL QIAGEN Proteinase K and gently invert the tube 5 times.
    ii. Incubate at 50°C for 2 h and gently inverting the tube 5 times after 1 h.
    iii. 10 min before the end of the lysis incubation time, prepare a Genomic-tip by equilibrating it with 4 mL Buffer QBT.
  e. Isolate DNA.
    i. Vortex the lysed sample for 5–10 s at maximum speed.
    ii. Gently pour the sample into five 2 mL tubes.
    iii. Centrifuge at 5,000 × *g* for 10 min at 4°C.
    iv. Immediately after the centrifugation pour the samples on the equilibrated Genomic-tip.
    v. Wash twice with 7.5 mL Buffer QC.
    vi. Pre-warm 5 mL Buffer QF to 50°C.
    vii. Eluted DNA with the 5 mL warm Buffer QF into a 50 mL tube.
  f. Concentrate DNA.
    i. Add 3.5 mL isopropanol to the sample.
    ii. Gently invert 10–20 times.
    iii. Pour sample into 2.0 mL tubes and centrifuged immediately at 10,000 × *g* for 30 min at 4°C.
    iv. Decant supernatant off and washed the pellet twice with 1 mL cold 70% ethanol.
    v. Centrifuge at 10,000 × *g* for 5 min at 4°C.
    vi. Air dry DNA for 5–10 min.
    vii. Re-suspend in 50 μL elution buffer.
    viii. Incubate at 37°C for 30–60 min and leave in the fridge for 16 h (overnight).

**CRITICAL:** It is important to not pipette the DNA, when possible, pour the DNA-containing liquid over to new vessels or use wide-bore, low-bind pipette tips.

**CRITICAL:** Use tongs when working with samples proximal to N_2_.

### High molecular weight DNA preparation quality control

**Timing: 2 days**

Quality control is the most important part of high molecular weight DNA preparation. It is important to start with high-quality DNA because the library preparation will result in some DNA fragmentation.

5. DNA concentration NanoDrop.
  a. Gently mix DNA sample.
  b. Measure the concentration with NanoDrop.
    ***Note:*** The A_260/280_ and A_260/230_ are important because they can help indicated if there are impurities, particularly residual phenol that may interfere with the sequencing. The values should be approximately A_260/280_ 1.80 and A_260/230_ should be 2.0–2.2 (see the Thermo Fisher Scientific NanoDrop T042-TECHNICAL BULLETIN).
6. DNA concentration using Qubit.
  a. Obtain the DNA concentration using the standard Qubit protocol.
    ***Note:*** Make sure to perform the calibration.
  b. The concentration should be in a similar range to the NanoDrop.
    ***Note:*** If the concentration measurements are off by over 0.5-fold then there may be impurities in your sample that can interfere with Nanopore sequencing.
7. Pulse-field gel electrophoresis, with Pippin Pulse.
  a. Cast 0.75% high strength agarose gel.
    i. 0.825 g of agarose in 0.5% KBB buffer (diluted from 10× KBB stock with ultra-pure water).
      ***Note:*** TBE buffer can also be used, see Pippin Pulse User Manual.
  b. Prepare samples.
    i. 100 ng of DNA in 10 μL of elution buffer with 2 μL of loading buffer.
  c. Place gel with the correct orientation in tank, fill with 0.5% KKB.
  d. Load 10 μL samples and 5 Kb ladder at both ends of the gel.
  e. Run on the program for 5–80 Kb of DNA at 75 V for 15 h (overnight).
  f. Soak gel in ethidium bromide for 30 min.
  g. Image gel.
    ***Note:*** This step will indicate how fragmented the DNA is and is important for decided to go forward with the sequencing.
    **CRITICAL:** Caution when handling ethidium bromide, it is a potential carcinogen.

### Long-read nanopore sequencing

**Timing: 2–3 days**

Followed the protocol provided by Nanopore.

8. Library preparation.
  a. Prepare the samples for sequencing using the Nanopore ligation protocol provided with the current version.

***Note:*** We used SQK LSK-109 for the parthenogenetic *D. mercatorum* genome and SQK LSK-110 for the sexually reproducing *D. mercatorum* genome.

9. ***Note:*** Optional Agilent TapeStation.
  a. Run library on the TapeStation to ensure it is not fragmented after the library is prepared.
10. Prepare flow cell and start sequencing.
  a. Prime flow cell for sequencing with the protocol provided for the flow cell.
  b. Load sample on the flow cell and initiated the sequencing run.

### Short-read DNA library preparation and sequencing

**Timing: 2 days**

A common step in preparing genome assemblies is polishing the assembly with short-read genome data produced using another method. Hi-C is most often used for polishing because it will give a better chromosome-level assembly. However, for *Drosophila* it is possible to use short-read libraries since the chromosome arms are conserved.^21^^,^^22^^,^^23^^,^^24^ Hi-C is still recommended for obtaining better resolution of the non-coding regions of *Drosophila* genomes. Here we use Illumina sequencing technology to sequence a whole genome DNA preparation in combination with transcriptomics data to polish the genome assembly. The same respective methods to prepare genomic DNA for long-read sequencing were used for the short-read DNA preparation. However, for the short-read library preparation only a single fly was used for the genomic DNA preparation.

**Figure 2:**
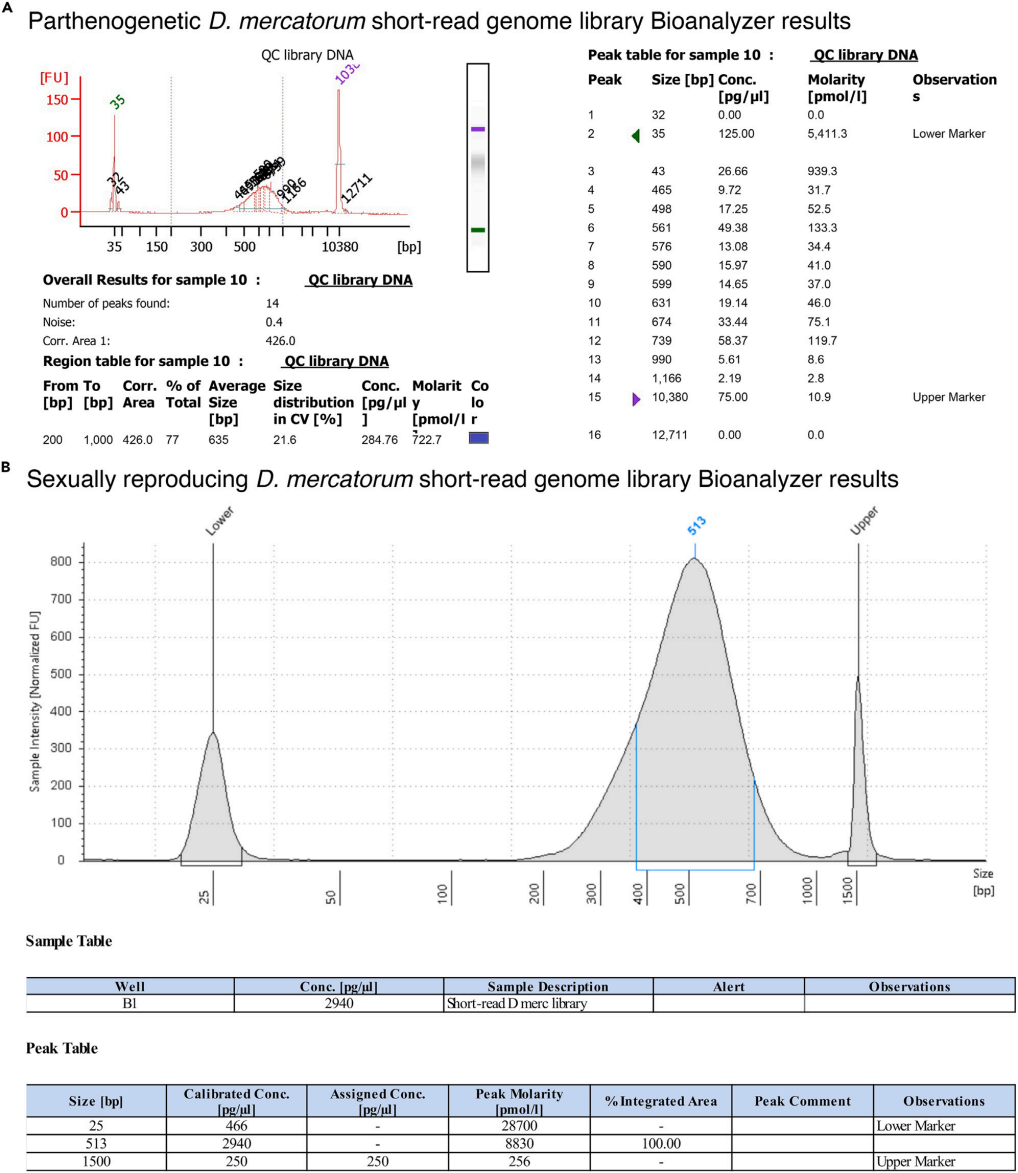
Short-read *D. mercatorum* genome library quality control (A) Bioanalyzer results for the parthenogenetic genome library. There is a single sample peak along with the lower and upper markers. The average size of the library is 635 bp. (B) TapeStation results for the sexually reproducing genome library. There is a single sample peak along with the lower and upper markers. The average size of the library is 513 bp.

***Note:*** We recommend using the TapeStation if possible due to the ease of use and longer lifetime of the reagents.

11. Library preparation can be done with almost any commercially available kit.
  ***Note:*** We used the KAPA HyperPrep Kits for NGS DNA Library Prep by Roche for the parthenogenetic genomes and the NEBNext Ultra II DNA Library Prep Kit for Illumina by New England Biolabs for the sexually reproducing genome.
  a. Follow the manufactures protocol in preparing the libraries.
12. Library quality control (the same as for high molecular weight DNA preparation).
  a. Quantify concentration using NanoDrop.
  b. Quantify concentration using Qubit.
  c. Check the quality of the library Agilent TapeStation or Agilent Bioanalyzer (Figure 2)

13. Sequencing can be done on any of the Illumina platforms.

***Note:*** We used both the MiSeq and the NovaSeq.

***Note:*** For our experiments we also polished the genome using transcriptomics data from our cells of interest. This was beneficial for comparison purposed between our genes of interest.

### Genome assembly and assessment

**Timing: 5 days – on a high-performance cluster**

This summarizes steps required to build and evaluate *Drosophila mercatorum* genome assemblies. The same process was used for both the sexually reproducing and parthenogenetic *Drosophila mercatorum* genomes. After the initial assembly these two genomes were compared to each other and to the *Drosophila melanogaster* reference genome. All the code used to perform the analysis is also publicly available on GitHub (see Data and code availability section).

14. Assemblies.
  a. Generate the assemblies with wtdbg2,^2^ minimap2^3^, and Samtools^4^ from the Nanopore data. Troubleshooting 4.
    # Sexually_reproducing = Sample_1 and Parthenogenetic = Sample_2
    # the same command were performed for each sample
    wtdbg2 -x ont -g 200m -i ../Sample_1.ont.fq.gz -t 24 -fo Sample_1.ont.wtdbg2
    wtpoa-cns -t 24 -i Sample_1.ont.wtdbg2..ctg.lay.gz -fo Sample_1.ont.wtdbg2.raw.fa
    minimap2 -t 24 -ax map-ont -r2k Sample_1.ont.wtdbg2.raw.fa ../Sample_1.ont.fq.gz ∖
     | samtools sort -@4 > Sample_1.ont.bam && samtools view -F0x900 Sample_1.ont.bam ∖
     | wtpoa-cns -t 24 -d Sample_1.ont.wtdbg2.raw.fa -i - -fo Sample_1.ont.wtdbg2.cns.fa
  b. Polish using Illumina data from the same isolate.
    i. Perform the alignment.
      bwa mem -t 48 Sample_1.ont.wtdbg2.cns.fa Sample_1/illumina/Sample_1.s_1.r_1.fq.gz
      Sample_1/illumina/ Sample_1.s_1.r_1.fq.gz >Sample_1.illumina.sam
      samtools view -b Sample_1.illumina.sam > Sample_1.illumina.raw.bam
      sambamba sort -t 48 -o Sample_1.illumina.bam Sample_1.illumina.raw.bam
      rm Sample_1.illumina.sam Sample_1.illumina.raw.bam
    ii. Variant calling using freebayes.^12^
      cat Sample_1.ont.wtdbg2.cns.fa.fai | cut -f 1 | parallel 'freebayes -f Sample_1.ont.wtdbg2.cns.fa --region {} Sample_1.illumina.raw.bam | bcftools view --no-version -Ob -o - >calls/{}.polish1.bcf'
    iii. Concatenate the variant calls.
      cat Sample_1.ont.wtdbg2.cns.fa.fai | cut -f 1 | while read f; do echo calls/$f.polish1.bcf; done > concat_list.txt
      bcftools concat -nf concat_list.txt | bcftools view -Ou -e 'type="ref"' | bcftools norm -Ob -f Sample_1.ont.wtdbg2.cns.fa -o Sample_1.illumina.bcf
    iv. Apply the polishing with bcftools consensus.^13^
      bcftools consensus -I 'QUAL>1 && (GT="AA" || GT="Aa")' -Hla -f Sample_1.ont.wtdbg2.cns.fa Sample_1.illumina.bcf > Sample_1.ont.wtdbg2.cns.polish.fa
      ***Note:*** Polished chromosome-level genome assemblies were created, and the assembly quality was assessed using standard metrics of NG50, coverage, and genome size (Table 1), all of which indicated that the genome sequences were of similar or greater quality than other *de novo Drosophila* genome assemblies. The apparent larger size of the sexually reproducing genome, which shows high representation of repetitive sequence, likely reflects different DNA preparation methods resulting in the overall size of the contigs (NG50) being larger.
      **Table 1:** Sexually reproducing and parthenogenetic genome size, coverage, contig number, continuity, and repeat content

		Sexually reproducing	Parthenogenetic
Genome size		171,182,504 bp	161,570,079 bp
Coverage		87.73×	95.74×
Contig number		526	330
Continuity	Contig Length (mean)	307,882 bp	489,606 bp
	Contig NG50	22,671,956 bp	16,356,382 bp
Repeat Content	Bases in repetitive regions	34,751,631 bp	29,808,408 bp
	Repetitive (%)	20.30%	18.45%
GC Content		40.65%	40.33%
15. Alignments to compare genomes *D. mercatorum* genomes to each other and to the *D. melanogaster* reference genome.
  a. Make alignments.
    fastix -p "Sample_1#" Sample_1.ont.wtdbg2.cns.polish.fa | bgzip -@ 24 >Sample_1.fa.gz && samtools faidx Sample_1.fa.gz
    fastix -p "Sample_2#" Sample_2.ctg.polished1.fa | bgzip -@ 24 > Sample2.fa.gz && samtools faidx Sample_2.fa.gz
    fastix -p "dmel6#" GCF_000001215.4_Release_6_plus_ISO1_MT_genomic.fna | bgzip -@ 24 >dmel6.fa.gz && samtools faidx dmel6.fa.gz
  b. Align genomes with wfmach.^7^
    wfmash -p 70 -t 48 dmel6.fa.gz Sample_1.fa.gz | gzip >dmel6_vs_Sample_1.26.paf.gz
    wfmash -p 70 -t 48 dmel6.fa.gz Sample_2.fa.gz | gzip >dmel6_vs_Sample_2.paf.26.gz
    wfmash -t 48 Sample_1.fa.gz Sample_2.fa.gz | gzip >Sample_1_vs_Sample_2.25.paf.gz
  c. Compute summary statistics on top of the outputs of the alignments.
    < f= Sample_1_vs_ Sample_2.25.paf.gz ; zcat $f | sed s/gi:f:// | awk '{ sum += $13 ∗ $10; tot += $10; block += $11; } END { print "matches",tot; print "block",block; print "gap.id", sum/tot; print "block.id",tot/block ∗ 100; }'
    < f=dmel6_vs_Sample_1.26.paf.gz ; zcat $f | sed s/gi:f:// | awk '{ sum += $13 ∗ $10; tot += $10; block += $11; } END { print "matches",tot; print "block",block; print "gap.id", sum/tot; print "block.id",tot/block ∗ 100; }'
    < f=dmel6_vs_Sample_2.26.paf.gz ; zcat $f | sed s/gi:f:// | awk '{ sum += $13 ∗ $10; tot += $10; block += $11; } END { print "matches",tot; print "block",block; print "gap.id", sum/tot; print "block.id",tot/block ∗ 100; }'
    ***Note:*** The comparison of the *D. mercatorum* genome assemblies with the *D. melanogaster* reference genome showed the contigs match to single chromosome arms (Figures 3A and 3B). The content of chromosome arms is largely conserved between *Drosophila* species.^21^^,^^22^^,^^23^^,^^24^ There was 75.5% (Figure 3A) and 75.8% (Figure 3B) gap-compressed sequence identity (n.b. this metric counts each gap as a single base irrespective of length, as described in http://lh3.github.io/2018/11/25/on-the-definition-of-sequence-identity) for the parthenogenetically and sexually reproducing genomes compared to the *D. melanogaster* genome, respectively. The alignment of the parthenogenetic genome covered 73.4 Mbp, while the sexually reproducing genome covered 75.3 Mbp of the *D. melanogaster* reference. The parthenogenetic and sexually reproducing *D. mercatorum* genomes were highly similar to each other, having 98.78% gap-compressed sequence identity (Figure 3C).
    **Figure 3:**
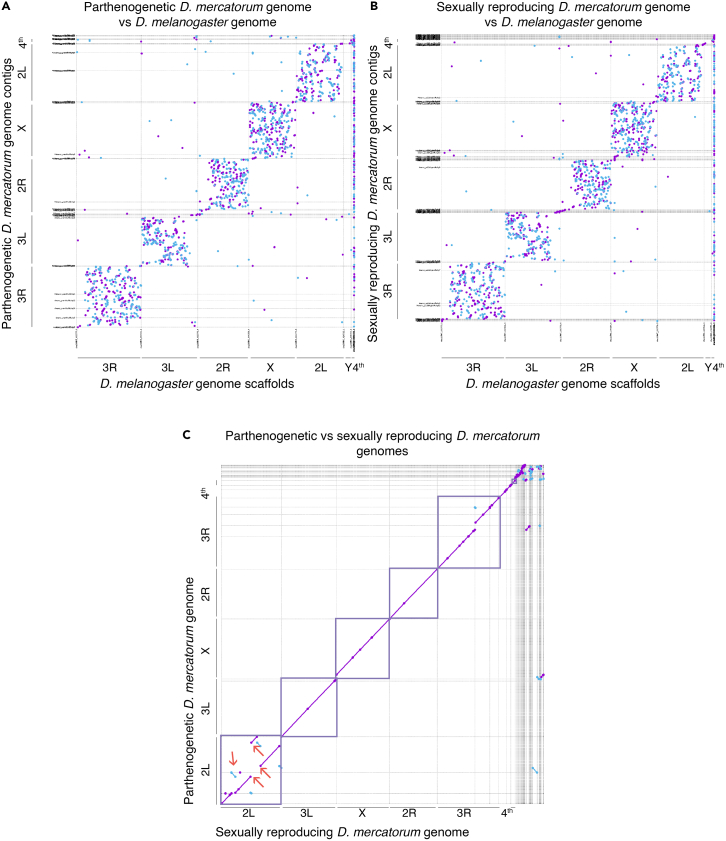
Genome assembly comparisons (A) Parthenogenetic *D. mercatorum* genome compared to the *D. melanogaster* reference genome (release 6). (B) Sexually reproducing *D. mercatorum* genome compared to the *D. melanogaster* reference genome (release 6). (C) Parthenogenetic *D. mercatorum* genome compared to the sexually reproducing *D. mercatorum* genome. Purple dots/lines represent sequences matching against the forward strand and blue the reverse. The red arrows indicate inversions. These images were originally published by Sperling et al.^1^
16. Coverage analysis with bedtools^8^ using the input Nanopore reads to measure the completeness of the assemblies.
  a. Map assemblies.
    # use the same commands for each assembly
    minimap2 -x map-ont -a -t 48 assemblies/Sample_1.fa.gz Sample_1.ont.fq.gz >Sample_1.ont.sam && ∖ samtools view -b Sample_1.ont.sam > Sample_1.ont.bam.1 &&∖ samtools sort -@ 24 -o Sample_1.ont.bam.1 && ∖ samtools index Sample_1.ont.bam && ∖ rm -f Sample_1.ont.bam.1 Sample_1.ont.sam
  b. Use samtools to get the coverage for the contigs.
    samtools coverage Sample_1.ont.bam >Sample_1.ont.bam.samtools_coverage.txt
    samtools coverage -w 160 Sample_1.ont.bam >Sample_1.ont.bam.samtools_coverage.asciiart.txt
  c. Get a summary of the coverage statistics, weighted on a per-base pair (not per-contig) level.
    < Sample_1.ont.bam.samtools_coverage.txt awk '{ len=$3-$2; tot += len; sum += len ∗ $6; } END { print sum / tot; }'
    **96.9121**
    < Sample_2.ont.bam.samtools_coverage.txt awk '{ len=$3-$2; tot += len; sum += len ∗ $6; } END { print sum / tot; }'
    **99.398**
  d. Retrieve the depth coverage.
    < Sample_1.ont.bam.samtools_coverage.txt awk '{ len=$3-$2; tot += len; sum += len ∗ $7; } END { print sum / tot; }'
    **79.2321**
    < Sample_2.ont.bam.samtools_coverage.txt awk '{ len=$3-$2; tot += len; sum += len ∗ $7; } END { print sum / tot; }'
    **93.8656**
    ***Note:*** For the parthenogenetic and sexually reproducing *D. mercatorum* genome assemblies there was uniform coverage for the larger contigs (Figure 4). However, the smaller contigs did show variation in coverage. This was more pronounced in the sexually reproducing *D. mercatorum* genome than the parthenogenetic *D. mercatorum* genome. This indicates that there may be haplotype specific contigs or that there may be genome contamination from one of the commensal organisms present in *Drosophila* lab cultures.
    **Figure 4:**
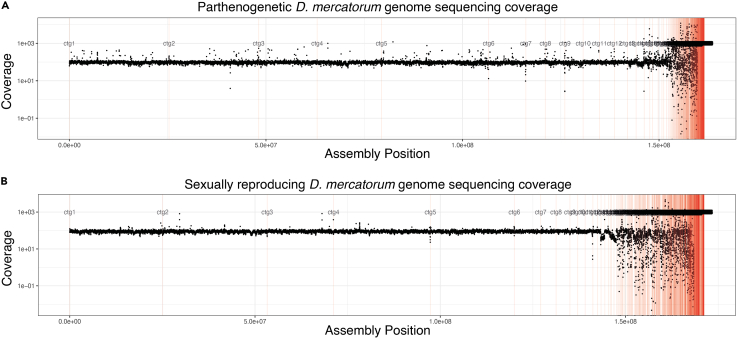
Genome coverage (A) coverage for the sexually reproducing *D. mercatorum* genome. (B) coverage for the sexually reproducing *D. mercatorum* genome. These images were originally published by Sperling et al.^1^
17. Self-heterozygosity analysis.
  a. Establish estimates for pairwise heterozygosity in each strain. We aligned the Illumina data used for polishing back to the assemblies.
    # use the same commands for each assembly
    bwa mem -t 24 assemblies/Sample_1.fa.gz Sample_1/illumina/Sample_1.s_1.r_1.fq.gz Sample_1/illumina/Sample_1.s_1.r_2.fq.gz > Sample_1.illumina.sam && samtools view -b Sample_1.illumina.sam > Sample_1.illumina.bam && sambamba sort -t 24 Sample_1.illumina.bam
  b. Call variants.
    freebayes -f assemblies/Sample_1.fa Sample_1.illumina.sorted.bam >Sample_1.illumina.vcf
  c. Normalize.
    < Sample_1.illumina.vcf awk '/ˆ#/ || NF == 10' | vcffilter -f 'QUAL > 20' | vcfwave | vcffilter -f 'TYPE = snp' > Sample_1.illumina.q20wave.vcf
  d. Measure heterozygosity.
    < % bcftools stats -s - Sample_1.illumina.q20wave.vcf | grep ˆPSC | cut -f 6
    **109035**
    < % bcftools stats -s - Sample_2.illumina.q20wave.vcf | grep ˆPSC | cut -f 6
    **16474**
    ***Note:*** An assembly quality value (QV) metric, based on comparison of 19-mers in the Illumina data to the assemblies, suggest error rates of around 1/1000; the QV for the sexually reproducing *D. mercatorum* genome is 26.545 and 28.6212 for the parthenogenetic *D. mercatorum* genome, which is adequate for our analyses. We estimated the pairwise heterozygosity with the Illumina data and found that within-strain variation was low. There were 109,035 heterozygous single nucleotide polymorphisms (SNPs) in the sexually reproducing *D. mercatorum* genome, and since the genome is 171,182,504 bp the pairwise heterozygosity is estimated at 0.0637% for SNPs. By contrast, there were 16,474 heterozygous SNPs in the parthenogenetic *D. mercatorum* genome, and since the genome is 16,1570,079 bp the pairwise heterozygosity is estimated at 0.0102% for SNPs.
18. Inter-strain divergence.
  a. Repeat the above analysis, but for each strain align against the other *D. mercatorum* assembly and then call variants.
    bwa mem -t 48 assemblies/Sample_2.fa.gz Sample_2/illumina/Sample_2.s_2.r_1.fq.gz
    Sample_2/illumina/Sample_2.s_2.r_2.fq.gz > Sample_2.vs_Sample_1.sam && samtools view -b
    Sample_2.vs_Sample_1.illumina.sam >Sample_2.vs_Sample_1.illumina.bam && sambamba sort -t 48 Sample_2.vs_Sample_1.illumina.bam && freebayes -f assemblies/Sample_1.fa
    Sample_2.vs_Sample_1.illumina.sorted.bam >Sample_2.vs_Sample_1.illumina.vcf
    bwa mem -t 48 assemblies/Sample_2.fa.gz Sample_1/illumina/Sample_1.s_1.r_1.fq.gz
    Sample_1/illumina/Sample_1.s_1.r_2.fq.gz >Sample_1.vs_Sample_2.illumina.sam && samtools view -b
    Sample_1.vs_Sample_2.illumina.sam >Sample_1.vs_Sample_2.illumina.bam && sambamba sort -t 48
    Sample_1.vs_Sample_2.illumina.bam && freebayes -f assemblies/Sample_2.fa Sample_1.vs_Sample_2.illumina.sorted.bam > Sample_1.vs_Sample_2.illumina.vcf
  b. Normalize.
    # use the same commands for each assembly
    < Sample_1.vs_ Sample_2.illumina.vcf awk '/ˆ#/ || NF == 10' | vcffilter -f 'QUAL > 20' | vcfwave | vcffilter -f 'TYPE = snp' >Sample_1.vs_Sample_2.illumina.q20wave.vcf
  c. Measure the number of non-reference homozygous SNPs (not heterozygous) for the parthenogen.
    < % bcftools stats -s - Sample_2.vs_Sample_1.illumina.q20wave.vcf | grep ˆPSC | cut -f 5
    **1442703**
    < % bcftools stats -s - Sample_1.vs_Sample_2.illumina.q20wave.vcf | grep ˆPSC | cut -f 5
    **1328517**
    ***Note:*** The genomes contained 0.82%–0.84% SNPs when compared to each other.
19. K-mer Spectrum Analysis using Meryl and Merqury^9^
  a. Build Meryl databases for the read sets.
    # use the same commands for each assembly
    zcat Sample_1/illumina/Sample_1.s_1.r_1.fq.gz Sample_1/illumina/Sample_1.s_1.r_2.fq.gz | pigz >
    Sample_1/illumina/Sample_1.s_1.r_both.fq.gz
    file=Sample_1/illumina/Sample_1.s_1.r_both.fq.gz meryl k=19 count output $file.meryl $file
  b. Run Merqury.
    merqury.sh Sample_1/illumina/Sample_1.s_1.r_both.fq.gz.meryl assemblies/Sample_1.fa Sample_1.merqury
  c. Plot the k-mer copy number spectra for both assemblies, using R
    x <- read.delim(‘Sample_1.merqury.Sample_1.spectra-cn.hist')
    x$Copies <- as.factor(x$Copies)
    ggplot(x, aes(x=kmer_multiplicity, y=Count, color=Copies)) + geom_line() + coord_cartesian(ylim=c(0,6.5e6), xlim=c(0,100))
    ***Note:*** There were no haplotype specific contigs as shown by the single peak present in the k-mer multiplicity plots (Figure 5).

**Figure 5:**
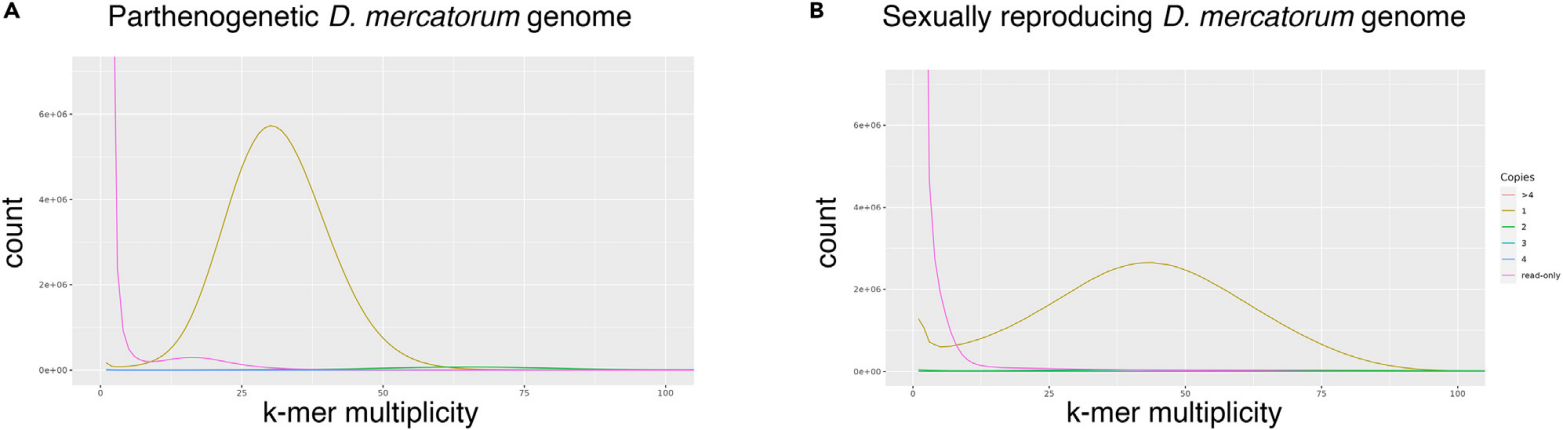
Haplotype analysis (A and B) Parthenogenetic and Sexually reproducing *D. mercatorum* genomes 19 bp-kmer copy number spectra showing a single homozygous peak (1× in assembly, and average read depth in the Illumina data). These images were originally published by Sperling et al.^1^

### Genome annotation and assessment

**Timing: 5 days – on a high-performance cluster**

This summarizes steps required to annotate and evaluate *Drosophila mercatorum* genome assemblies. After the below steps are complete the genome is otherwise ready for functional work and any transcriptomics data can be mapped. However, we also used our transcriptomics data to aid in refining the annotation at the loci of interest. The same process was used for both the sexually reproducing and parthenogenetic *Drosophila mercatorum* genomes we described above. All the code used to perform the analysis is also publicly available on GitHub (see Data and code availability section).

20. Identify repetitive sequences in the genome using RepeatModeler^10^ and masking them using RepeatMasker^11^
  a. Call repeats *de novo* using RepeatModeler.
    bwa index Sample_2.fa
    RepeatModeler -database Dmerc -LTRStruct -pa 6
  b. Mask *D. mercatorum* assembly using RepeatMasker.
    ***Note:*** Hard-masking completely removes repetitive sequences whereas soft-masking keeps them in the file but indicates the repeats are there. We used soft masking for the annotation and hard-masking later in the protocol in Step 25a.
    # Hard-masked genome
    singularity exec -B TandemRepeatFinder/trf:/opt/trf:ro RepeatMasker -gccalc -s -nolow -norna -gff -pa 25 -lib Dmerc-families.fa Sample_2.fa
    # Soft-masked genome
    singularity exec -B TandemRepeatFinder/trf:/opt/trf:ro RepeatMasker -xsmall -gccalc -s -nolow -norna -gff -pa 25 -dir repmasker_class_SOFTMASK -lib Dmerc-families.fa Sample_2.fa
21. Annotation to get the *ab initio* gene prediction.
  a. Trim raw reads using Cutadapt.^18^
    for i in {2..10}
    do
     if [ $i -lt 10 ]
      then
      echo "1-9: Sample_${i}.s_2.r_1.fq.gz"
      cutadapt -j 20 -a AGATCGGAAGAGC -A AGATCGGAAGAGC -q 20 --minimum-length 50 -o trimmed/Sample_${i}.s_2.r_1.fq.TRIM.gz -p trimmed/Sample_${i}.s_2.r_2.fq.TRIM.gz
    Sample_${i}.s_2.r_1.fq.gz Sample_${i}.s_2.r_2.fq.gz
     else
      echo "10: Sample_${i}.s_2.r_1.fq.gz"
      cutadapt -j 20 -a AGATCGGAAGAGC -A AGATCGGAAGAGC -q 20 --minimum-length 50 -o trimmed/Sample_${i}.s_2.r_1.fq.TRIM.gz -p trimmed/Sample_${i}.s_2.r_2.fq.TRIM.gz
    Sample_${i}.s_2.r_1.fq.gz Sample_${i}.s_2.r_2.fq.gz
     fi
    done
  b. Prepare *D. mercatorum* fasta for STAR^12^ mapping.
    # Edit header of fasta
    sed 's/ len.∗//g' Sample_2.fa > Sample_2.fa_headerEdit
    # Prepare genome for STAR
    mkdir STAR_genome_dir_headerEdit
    STAR-2.7.0e/source/STAR --runThreadN 15 --runMode genomeGenerate --genomeDir
    STAR_genome_dir_headerEdit --genomeFastaFiles Sample_2.fa_headerEdit
  c. Map with STAR.
    echo $file
    fileR2="$(echo $file | sed 's/r_1/r_2/g')"
    outname="$(echo $file | sed 's/r_1∖.//g')"
    echo "$fileR2"
    echo "headerEditMap_Unmasked/${outname%.fastq.gz}"
    echo "$outname"
    STAR --runThreadN 25 --genomeDir STAR_genome_dir_headerEdit --readFilesIn $file $fileR2 --readFilesCommand zcat --outFilterMultimapNmax 10 --outReadsUnmapped FastX --outSAMstrandField intronMotif --outSAMtype BAM SortedByCoordinate --outFileNamePrefix
    headerEditMap_Unmasked/${outname%.fastq.gz}
    done
  d. Merge bam files using Samtools.
    samtools merge unmasked_map.bam Sample_2002Aligned.sortedByCoord.out.bam
    Sample_2003Aligned.sortedByCoord.out.bam Sample_2004Aligned.sortedByCoord.out.bam
    Sample_2005Aligned.sortedByCoord.out.bam Sample_2006Aligned.sortedByCoord.out.bam
    Sample_2007Aligned.sortedByCoord.out.bam Sample_2008Aligned.sortedByCoord.out.bam
    Sample_2009Aligned.sortedByCoord.out.bam Sample_2010Aligned.sortedByCoord.out.bam
  e. *Ab initio* gene prediction with BRAKER2.^13^ Troubleshooting 5.
    braker.pl --species=Dmerc _merge_unmasked_soft --genome=
    repmasker_class_SOFTMASK/Sample_2.fa.masked_headerEdit --softmasking --
    bam=unmasked_map.bam --cores 12
    ***Note:*** The number of genes predicted by BRAKER2 (Table 2).
    **Table 2:** Genome completeness for the parthenogenetic and sexually reproducing *D. mercatorum* genomes

		Sexually reproducing	Parthenogenetic
Functional completeness	Predicted genes (Breaker2)	17,364	17,566
	Uniquely mapped reads (STAR)	80%–91%	74%–89%
	BUSCO	98.00%	99.00%
22. Link the BRAKER2 *de novo* annotations with the *D. melanogaster* annotations.
  a. CDS extraction from BRAKER.gtf.
    gffread -g Sample_2.fa -x dmerc.BRAKER_CDS_gffread.fasta braker.gtf
    esearch -db nuccore -query "Drosophila melanogaster [organism]" | efetch -db nuccore -format uid > Dmel_gilist_July2020.gi
    esearch -db protein -query "Drosophila melanogaster [organism]" | efetch -db protein -format uid > Dmel_gilist_protein_July2020.gi
    esearch -db protein -query "Drosophila [organism]" | efetch -db protein -format uid >
    All_Drosophila_gilist_proteins_Sept2020.gi
    update_blastdb.pl --showall [∗]
    update_blastdb.pl --decompress nr
  b. BLASTx^14^ the extracted CDS vs. the *D. melanogaster* protein databank.
    blastx -query dmerc.BRAKER_CDS_gffread.fasta -db nr -num_threads 60 -outfmt '6 qseqid stitle qlen slen length qcovs qcovhsp evalue bitscore pident sacc sseqid sscinames' -gilist Dmel_gilist_protein_July2020.gi -max_target_seqs 137871 -out dmerc.BRAKER_CDS_gffread_BLASTx.txt
    sed 's/ /_/g' dmerc.BRAKER_CDS_gffread_BLASTx.txt | sed 's/∖,//g' | sed 's/∖_∖[Drosophila∖_melanogaster∖]//g' | cut -f1,2,3,4,5,6,7,8,9,10,11,12 > dmerc.BRAKER_CDS_gffread_BLASTx_edit.txt
  c. Add Flybase gene name to the blast hits mapping file.
    i. Download and edit Flybase gene names file from Flybase.
      wgetftp://ftp.flybase.net/releases/current/precomputed_files/genes/fbgn_NAseq_Uniprot_fb_2020_03.tsv.gz
      zcat fbgn_NAseq_Uniprot_fb_2020_03.tsv.gz > fbgn_NAseq_Uniprot_fb_2020_03.tsv
      sed 's/## fbgn_NAseq_Uniprot_fb_2020_03.tsv | grep -v 'ˆ#' | sed 's/primary_FBgn∖#/primary_FBgn/g' | sed 's/∖t/,/g' > fbgn_NAseq_Uniprot_fb_2020_03_edit.csv
      Rscript fbgn_accession_file_edit_2020_03.R # to get fbgn_NAseq_Uniprot_fb_2020_03_edit_final.txt (see scripts folder on github)
    ii. Filter the BLAST output and add the Flybase gene names.
      Rscript add_fbgn_to_blast_strict_pident35.R 30 all_gene_ids.txt
      dmerc.BRAKER_CDS_gffread_BLASTx_edit.txt fbgn_NAseq_Uniprot_fb_2020_03_edit_final.txt
      fbgn2blast_out_strict_pident35/
  d. Reverse BLAST the *D. melanogaster* CDS to *D. mercatorum* using tBLASTx.
    # Makeblastdb
    cd blast/
    mkdir blast/Dmerc_CDS
    makeblastdb -in dmerc.BRAKER_CDS_gffread.fasta -title Dmerc_CDS -dbtype nucl -out blast/Dmerc_CDS -hash_index
    blastdbcmd -db blast/Dmerc_CDS -info
    sed 's/ type.∗//g' dmel-all-CDS-r6.35.fasta > dmel-all-CDS-r6.35_edit.fasta
    tblastx -query dmel-all-CDS-r6.35_edit.fasta -db blast/Dmerc_CDS -num_threads 20 -outfmt '6 qseqid stitle qlen slen length qcovs qcovhsp evalue bitscore pident sacc sseqid sscinames' -max_target_seqs 18313 -out mel_CDS_to_Dmerc_CDS_gffread_tBLASTx.txt
    cd Dmel_to_Dmerc_tblastx
    sed 's/ /∖_/g' mel_CDS_to_Dmerc_CDS_gffread_tBLASTx.txt | awk '$6>50 && $8<1e-10 && $10>35' | cut -f1,2 | sort | uniq | sed 's/∖_gene∖=/∖t/g' | cut -f1,3 | sort | uniq > mel_CDS_to_Dmerc_CDS_gffread_tBLASTx_qcov50_eval1e10_pident35.txt
    # Get forward & reverse orthologs
    Rscript create_tblastx_map_file.R # (see scripts folder on github)
23. STAR mapping with BRAKER.
  a. Map RNA-sequencing reads considering the BRAKER.gtf file.
    i. Create genome directory with braker.gtf.
      mkdir STAR_genome_dir_braker
    ii. Unmask reference braker.gtf with STAR.
      STAR --runThreadN 15 --runMode genomeGenerate --genomeDir
      STAR_genome_dir_braker --genomeFastaFiles
      Sample_2.fa_headerEdit --sjdbGTFfile
      braker.gtf --sjdbOverhang 149
    iii. Get the intron’s minimum and maximum.
      grep "intron" braker.gtf
      awk '$3=="intron"' braker.gtf | awk 'BEGIN{OFS="∖t"} NR >= 0 { $6 = $5 - $4 } 1' | sort -k6,6n | cut -f6 | head
      # 12
      awk '$3=="intron"' braker_mergeBam/braker.gtf | awk 'BEGIN{OFS="∖t"} NR >= 0 { $6 = $5 - $4 } 1' | sort -k6,6n | cut -f6 | tail
      # 302757
    iv. Map with STAR using the genome generated with BRAKER.gtf.
      for file in raw_reads/trimmed/∗r_1.fq.TRIM.gz
      do
      echo $file
      fileR2="$(echo $file | sed 's/r_1/r_2/g')"
      outname="$(echo $file | sed 's/r_1∖.//g')"
      echo "$fileR2"
      echo "${outname%.fastq.gz}"
      echo "$outname"
      STAR --runThreadN 25 --genomeDir STAR_genome_dir_braker --readFilesIn $file $fileR2 --readFilesCommand zcat --alignIntronMin 12 --alignIntronMax 302757 --outFilterMultimapNmax 10 --outReadsUnmapped FastX --outSAMstrandField intronMotif --outSAMtype BAM SortedByCoordinate --outFileNamePrefix mapped/${outname%.fastq.gz}
      done
    v. Check the number of uniquely mapped reads.
      for file in ∗Log.final.out
      do
      echo "$file"
      x=$(cat $file | grep "Uniquely mapped∖|Number of input reads")
      echo "$x"
      done
    vi. Index the bam files and sort by read name using Samtools.
      for file in ∗.out.bam
      do
      echo "READING: $file"
      outname="$(echo $file | sed 's/∖.sortedByCoord∖.out∖.bam/∖.sortedByName∖/g')"
      echo "$outname"
      samtools sort -n $file
      ${outname}
      samtools index $file
      done
    vii. FeatureCounts.^17^
      #multiple files into one readCount file
      featureCounts -p -a braker.gtf -o Sample_2002_to_Sample_2010_BrakerSoftMaskGTF.readCounts -t exon -g gene_id --extraAttributes gene_symbol -T 14 Sample_2002Aligned.sortedByName.bam Sample_2003Aligned.sortedByName.bam Sample_2004Aligned.sortedByName.bam
      Sample_2005Aligned.sortedByName.bam Sample_2006Aligned.sortedByName.bam
      Sample_2007Aligned.sortedByName.bam Sample_2008Aligned.sortedByName.bam
      Sample_2009Aligned.sortedByName.bam Sample_2010Aligned.sortedByName.bam
      done
24. Genes per contig distribution.
  #In bash: cat
  braker.gtf | cut -f1,9 | sed ‘s/gene∖_id//g’ | sed ‘s/transcript∖_id//g’ | awk ‘{print $1”∖t”$2}’ | sed ‘s/∖;//g’ | sed ‘s/∖”//g’ | sed ‘s/∖..∗//g’ | sort | uniq | cut -f1 | sort | uniq -c > genes_per_contig.txt
  #In R:
  gene_contig = read.table("genes_per_contig.txt",header=F)
  names(gene_contig) = c("Count","Contig")
  gene_contig = gene_contig[order(gene_contig$Count,decreasing = T),]
  gene_contig$Contig = factor(gene_contig$Contig,levels = gene_contig$Contig)
  a. Scaffold length.
    scafflen = read.table("dmerc.ctg.polished1_scaffold_length.txt") # see file in github repo
    scafflen = scafflen[order(scafflen$V2,decreasing = T),]
    names(scafflen) = c("Contig","length")
    # merge dfs
    gene_contig_length = merge(scafflen,gene_contig,by="Contig",all=T)
    gene_contig_length$Count[is.na(gene_contig_length$Count)] = 0
    gene_contig_length$Prop = gene_contig_length$Count / gene_contig_length$length
  b. Plot number of genes per contig using ggplot.
    p = ggplot(data = gene_contig,aes(x=Contig,y=Count)) + geom_bar(stat="identity") + theme_bw() +
     theme(axis.text.x = element_text(angle = 45, hjust = 1, size = 10,colour="black"),
      axis.text.y = element_text(size = 10,colour="black",hjust=1),
      axis.title = element_text(size = 12,colour="black")) +
     xlab("Contig") +
     scale_y_continuous(name="Count of genes") +
     scale_x_discrete(labels=gsub("ctg","",levels(gene_contig$Contig)))
    ggsave(filename = "Gene_count_contigs.pdf",p,height=4,width=10)
    ***Note:*** The majority of putative genes were distributed evenly on the 14 largest contigs of both sexually reproducing and parthenogenetic *D. mercatorum* genomes (Figure 6).
    **Figure 6:**
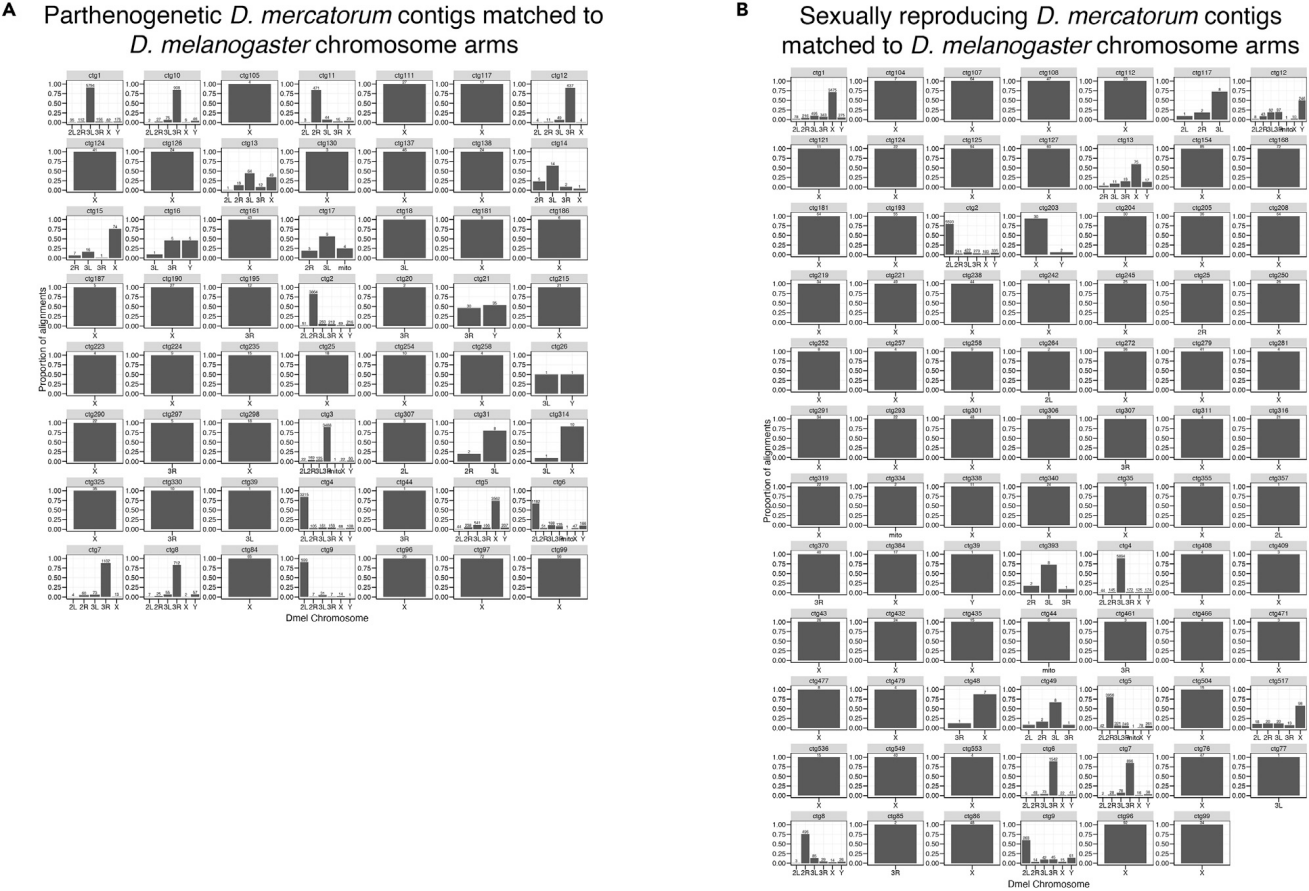
Predicted genes per contig (A) Contigs of the parthenogenetic *D. mercatorum* genome. (B) Contigs of the sexually reproducing *D. mercatorum* genome. These images were originally published by Sperling et al.^1^
25. Determine genes per contig by matching to *D. melanogaster* chromosomes using nucmer^15^
  a. Run nucmer on the hard-masked genome.
    nucmer rep_masked/dmel-all-chromosome-r6.27.fasta.masked Sample_2.masked_headerEdit --coords --maxgap 500 --maxmatch --prefix Dmerc_Dmel_hardmask_nucmer_maxmatch_maxgap500
  b. Analyze proportions.
    show-coords Dmerc_Dmel_hardmask_nucmer_maxmatch_maxgap500.delta -l -c > Dmerc_Dmel_hardmask_nucmer_maxmatch_maxgap500_full.coords
  c. Subset to interesting chromosome arms using nucmer.
    nucmer.counts.edit1 = nucmer.counts.edit[nucmer.counts.edit$Dmel_Chr %in% c("2L","2R","3L","3R","X","Y","mitochondrion_genome"),]
    nucmer.counts.edit1$Dmel_Chr = gsub("mitochondrion_genome","mito",nucmer.counts.edit1$Dmel_Chr)
  d. Count total alignments.
    total = as.data.frame(tapply(nucmer.counts.edit1$Counts,nucmer.counts.edit1$Dmerc_Chr,sum))
    total1 = cbind(row.names(total),total)
    names(total1) = c("Dmerc_Chr","Total_Align")
  e. Merge.
    nucmer.counts.edit2 = merge(nucmer.counts.edit1,total1,by="Dmerc_Chr")
    nucmer.counts.edit2$PropAlign = nucmer.counts.edit2$Counts / nucmer.counts.edit2$Total_Align
  f. Plot with ggplot.
    Dmel_chr_plot = ggplot(nucmer.counts.edit2, aes(x = Dmel_Chr, y = Counts)) + geom_bar(stat="identity") + THEME_DEF_small +
     labs(x = "Dmel Chromosome", y = "Number of alignments") +
     facet_wrap(∼Dmerc_Chr, ncol= 7,scales = "free") +
     theme(strip.text = element_text(size = 17),
      axis.title.y = element_text(vjust=0.5,size=25),
      axis.title.x = element_text(vjust=-0.2,size=25),
      axis.text.x = element_text(size=18, colour = "black"),
      axis.text.y = element_text(size=18, colour = "black"))
    ggsave(Dmel_chr_plot, file="Dmerc_Contigs_to_Dmel_Chrom_Nucmer_maxgap500.pdf", height=20, width=20)
    Dmel_chr_plot_prop = ggplot(nucmer.counts.edit2, aes(x = Dmel_Chr, y = PropAlign)) + geom_bar(stat="identity") + THEME_DEF_small +
     labs(x = "Dmel Chromosome", y = "Proportion of alignments") +
     geom_text(aes(label=Counts), vjust=-0.2, color="black", position = position_dodge(0.9), size=3.5) +
     scale_y_continuous(name="Proportion of alignments", limits=c(0, 1.05)) +
     facet_wrap(∼Dmerc_Chr, ncol= 7,scales = "free") +
     theme(strip.text = element_text(size = 17),
      axis.title.y = element_text(vjust=0.5,size=25),
      axis.title.x = element_text(vjust=-0.2,size=25),
      axis.text.x = element_text(size=18, colour = "black"),
      axis.text.y = element_text(size=18, colour = "black"))
    ggsave(Dmel_chr_plot_prop, file="Dmerc_Contigs_to_Dmel_Chrom__Proportion_Nucmer_maxgap500.pdf", height=20, width=20)
  g. Select contigs based on major unique hit >40% (i.e., no other hits 40% or more).
    nucmer.counts.edit2.40perc = nucmer.counts.edit2[nucmer.counts.edit2$PropAlign >=0.40,]
    nucmer.counts.edit2.40perc[nucmer.counts.edit2.40perc$Dmerc_Chr %in%
    nucmer.counts.edit2.40perc$Dmerc_Chr[duplicated(nucmer.counts.edit2.40perc$Dmerc_Chr)],]
    nucmer.counts.edit2.40perc.uniq =
    nucmer.counts.edit2.40perc[!nucmer.counts.edit2.40perc$Dmerc_Chr %in%
    nucmer.counts.edit2.40perc$Dmerc_Chr[duplicated(nucmer.counts.edit2.40perc$Dmerc_Chr)],]
    nucmer.counts.edit2.40perc.uniq =
    nucmer.counts.edit2.40perc.uniq[order(nucmer.counts.edit2.40perc.uniq$Total_Align,decreasing = T),]
    ***Note:*** The matching of *D. mercatorum* contigs to *D. melanogaster* chromosome arms was done with k-mers using nucmer and the contigs largely matched to a single chromosome arm (Figure 7).
    **Figure 7:**
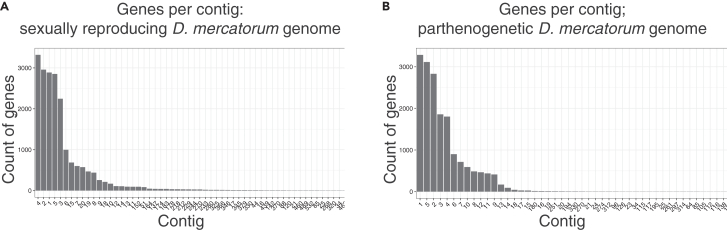
The genome assembly contigs are restricted to specific chromosome arms (A) Chromosome arm assignment to the contigs of the parthenogenetic *D. mercatorum* genome based on k-mer mapping using nucmer. (B) Chromosome arm assignment to the contigs of the sexually reproducing *D. mercatorum* genomes based on k-mer mapping using nucmer. These images were originally published by Sperling et al.^1^
26. Genome completeness estimated with BUSCO^16^ scores.

busco -i input_genome.fa -m genome -download_path path_to_download_dir -auto-lineage-euk -o input_genome_name -out_path path_to_output_dir -c 30

setwd("reference/nucmer_output/")

nucmer = read.table("Dmerc_Dmel_hardmask_nucmer_maxmatch_maxgap500_full_edit.coords",header=T)

# manual edit of above file to allow loading in R

nucmer$ID = paste(nucmer$Dmerc_Chr,"_",nucmer$Dmel_Chr,paste="")

nucmer.counts = as.data.frame(sort(table(nucmer$ID),decreasing = T))

nucmer.counts.edit = cbind(matrix(unlist(strsplit(as.character(nucmer.counts$Var1)," _ ")),ncol=2,byrow=T),nucmer.counts)

names(nucmer.counts.edit) = c("Dmerc_Chr","Dmel_Chr","ID","Counts")

nucmer.counts.edit$Dmerc_Chr = gsub(" ","", nucmer.counts.edit$Dmerc_Chr)

nucmer.counts.edit$Dmel_Chr = gsub(" ","", nucmer.counts.edit$Dmel_Chr)

nucmer.counts.edit$ID = gsub(" _ ","/", nucmer.counts.edit$ID)

nucmer.counts.edit$ID = gsub(" ","", nucmer.counts.edit$ID)

**Figure 8:**
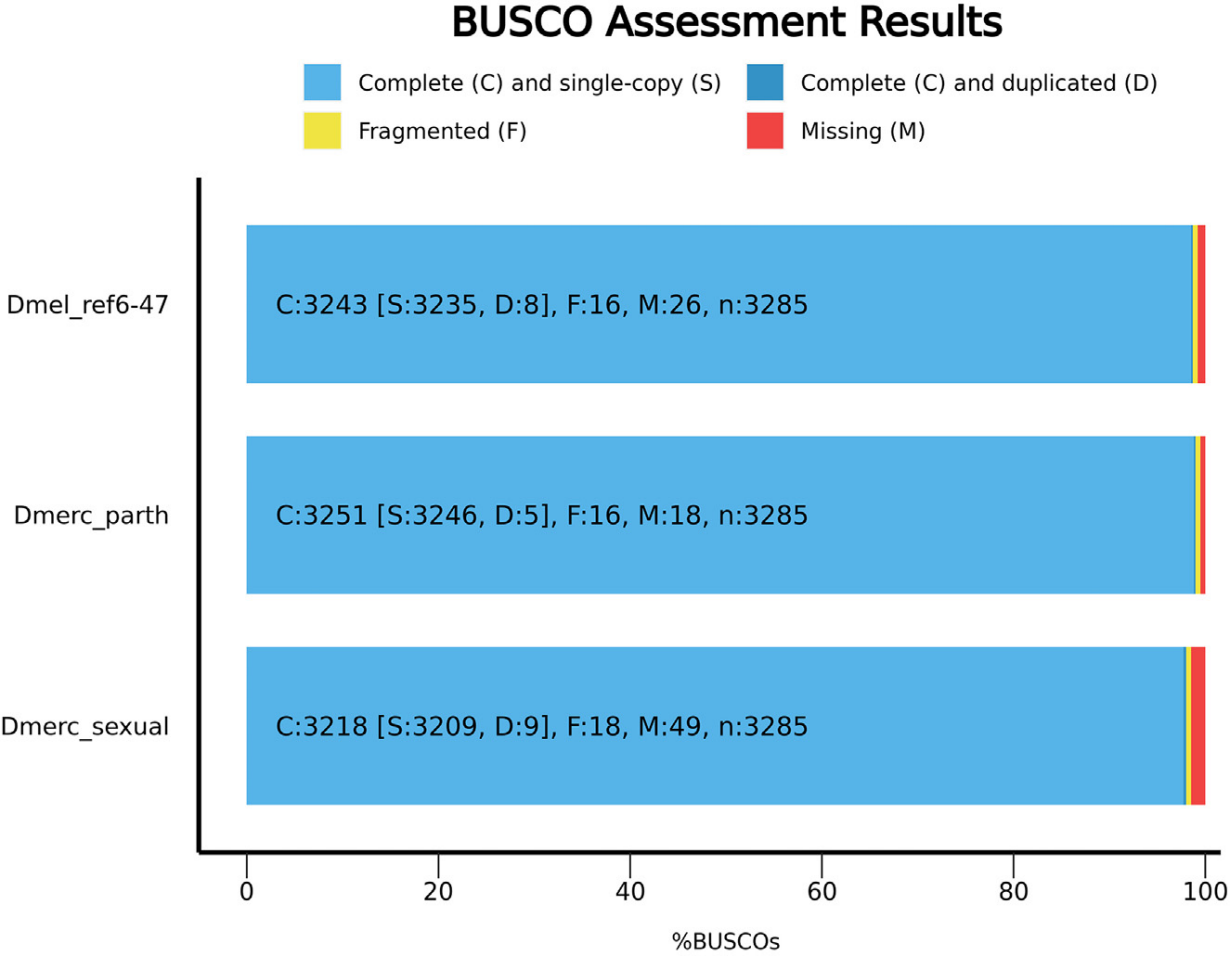
Assessment of genome assembly completeness by comparison against the Diptera specific BUSCO dataset for *D. melanogaster* as the control and the parthenogenetic and sexually reproducing *D. mercatorum* genome assemblies This image was originally published by Sperling et al.^1^

***Note:*** The genomes of the sexually reproducing and parthenogenetic *D. mercatorum* strains were 98% and 99% complete, respectively, in comparison to the dipteran-specific BUSCO dataset (Table 2; Figure 8). By comparison, the *D. melanogaster* reference genome (release 6) is only 98.7% complete when compared to the dipteran-specific BUSCO dataset. Together these analyses confirmed the expected high divergence between *D. melanogaster* and *D. mercatorum* and indicated that the chromosome-level genome assemblies for the sexually and parthenogenetically reproducing *D. mercatorum* strains were suited to comparison with the *D. melanogaster* genome for the purpose of identifying gene homologues.

## Expected outcomes

Both methods for DNA preparation will yield a highly complete genome that can be used as a foundation for functional genomics. The same number/weight of flies were used for each high molecular weight DNA preparation method, and 457.2 ng of DNA isolated using the phenol method and 2.57 μg using the Genomic-tip method. After the genomes were assembled and analyzed we achieved high quality chromosome-level genome assemblies (Table 1). They assembly quality assessed using standard metrics of NG50, coverage, and genome size, all of which indicated that the genome sequences were of similar or greater quality to other *de novo* *Drosophila* genome assemblies. The apparent larger size of the sexually reproducing genome, which shows high representation of repetitive sequence, likely reflects different DNA preparation methods resulting in the overall size of the contigs (NG50) being larger.

## Limitations

The limitations of this protocol are that the annotations are not precise, therefore requiring manual annotation. It is very important to manually verify the annotations prior to any functional work. This is relatively easy in *Drosophila*, since there is synteny between the chromosome arms and even the order of the genes in distantly related species.

## Troubleshooting

### Problem 1

**Phenol carryover (related to Step 2ai)**: phenol will interfere with Nanopore sequencing and hence the final preparation of your DNA preparation should not have any phenol.

### Potential solution

We avoided this by leaving some of the aqueous phase behind and not taking anything near the interphase of the two layers for each stage of the extraction. This was possible because we had high quantity of DNA. With more precious samples this may not be possible and therefore an additional extraction may be helpful in isolating more DNA. We also tried using QIAGEN phase lock tubes but found that the waxy phase lock layer did not separate the layers properly for our samples and thus resulted in additional handling of the DNA. Our resulting samples were of lower quality. Different types of samples may work better with phase lock tubes.

### Problem 2

**DNA heating (related to Step 2dvii)**: DNA is less stable when heated.

### Potential solution

We heated our sample at body temperature for a limited time in order to avoid leaving it to dry for 16 h (overnight) at 21°C (room temperature), which in our hands resulted in lower molecular weight samples. The heating may cause the DNA to break, therefore it is best to check if this heating is a problem for one’s samples and balance this against the conditions of the lab overnight (ambient temperature and exposure to contaminants).

### Problem 3

**Fly ice cube (related to Step 3bii)**: the broken-up pieces of flies form an ice cube instead of a powder.

### Potential solution

This is caused by condensation forming on the flies as they are being blended. This should be avoided because this can rupture the nuclei and break apart the DNA. In order to avoid this one must work quickly and keep the tube with the flies in contact with the liquid N_2_ for the entire duration of the blending process.

### Problem 4

**Bad assembly (related to Step 14a)**: An assembly with metrics that are much lower or higher than expected.

### Potential solution

We used wtdbg2 as our assembler, we did not get good results when we used our more often used assembler, Shasta.^25^ Therefore, we suggest trying a few since the results may be different depending on the repetitive content of the genomes you are assembling and the type of genome the assembler was designed for. We ended up choosing an assembler that gave us a more contiguous assembly and then we physically mapped the contigs using *in* *situ* hybridization and checked the chromosome arm content against *D. melanogaster* in order to be confident that the assembly was correct.^1^

### Problem 5

**Mixed assembly (related to Step 21e)**: We found that the sexually reproducing genome assembly contained contigs at lower-than-expected coverage, which we suspected to be non-*Drosophila* DNA. There were small contigs that were densely packed with genes, which is not typical of metazoan genomes. The *D. mercatorum* genomic DNA was prepared from whole animals and therefore potentially included the genome commensal organisms.

### Potential solution

We used basic local alignment search tool (BLAST) to determine what organism(s) the genes on all the small contigs came from. There are other tools available (such as BlobTools), but this simple method was more than adequate for our purpose. We found a common commensal gut bacterium to be our only contaminant. There were more contigs from the commensal organism in the assembly that was prepared using the Genomic-tip method than with the phenol method. Therefore, to avoid this then the phenol extraction method is a better option.

## Resource availability

### Lead contact

David M. Glover (dmglover@caltech.edu).

### Technical contact

Alexis L Sperling (alb84@cam.ac.uk).

### Materials availability

This study did not generate new unique reagents.

### Data and code availability

All code is publicly available. The genome assembly, analysis, and quality control code are on https://github.com/ekg/drosophila. The annotation, transcriptomics analysis, and quality control code are on https://github.com/FabianDK/Dmerc. All raw and analyzed *D. mercatorum* genomic data is on the European Nucleotide Archive (ENA): PRJEB64421. The transcriptomics data is on ENA: PRJEB43100.
